# STAT3 mediates resistance of CD44^+^CD24^−/low^ breast cancer stem cells to tamoxifen *in vitro*

**DOI:** 10.7555/JBR.26.20110050

**Published:** 2012-04-18

**Authors:** Xiaoyan Wang, Guozhu Wang, Yi Zhao, Xiaoan Liu, Qiang Ding, Jingping Shi, Yin Ding, Shui Wang

**Affiliations:** a Department of Breast Surgery, the First Affiliated Hospital of Nanjing Medical University, Nanjing, Jiangsu 210029, China;; b Laboratory of Mesoscopic Chemistry and Department of Polymer Science and Engineering, College of Chemistry and Chemical Engineering, Nanjing University, Nanjing, Jiangsu 210093, China.

**Keywords:** STAT3, breast cancer, cancer stem cells, tamoxifen drug resistance

## Abstract

We sought to determine whether STAT3 mediated tamoxifen resistance of breast cancer stem cells *in vitro*. The capacities for mammosphere formation and STAT3 expression of CD44^+^CD24^−/low^ MCF-7 and MCF-7 were observed. The CD44^+^CD24^−/low^ subpopulation ratio and its sensitivity to adriamycin were analyzed in MCF-7 and TAM resistant (TAM-R) cells. Cell cycle, apoptosis, STAT3 and phospho-STAT3 changes were observed after treatment with tamoxifen. Small interference RNA-mediated knockdown of STAT3 in TAM-R cells was also performed. CD44^+^CD24^−/low^ MCF-7 showed higher capacities for mammosphere formation and STAT3 expression than total MCF-7. The CD44^+^CD24^−/low^ subpopulation was also upregulated in TAM-R cells with less sensitivity to adriamycin than MCF-7. Cell cycle changes, anti-apoptotic effects and STAT3 changes were also found. Meanwhile, the knock-down of STAT3 in TAM-R resulted in an increase in sensitivity to tamoxifen. It is concluded that STAT3 plays an essential role in breast cancer stem cells, which correlated with tamoxifen resistance.

## INTRODUCTION

Breast cancer is the most frequently diagnosed female carcinoma and the second leading cause of cancer death for women of all ages. As the most important drug used in endocrine therapy, particularly for estrogen-related breast cancer, tamoxifen reduces the relapse rate by 39% per year and the mortality rate by 31% per year[1]. Therefore, tamoxifen remains the therapeutic choice for all pre-menopausal estrogen-related breast cancer, sequential therapy for post-menopausal patients and patients who cannot tolerate aromatase inhibitors. However, drug resistance in endocrine therapy is still a challenging clinical problem, and the mechanisms underlying tamoxifen resistance, which probably develops through multiple pathways, are still unclear.

Breast cancer stem cells are defined as a subpopulation of breast cancer cells that can self-renew and differentiate into other types of cancer cells. These cells are scarce in tumors but 100-fold more tumorigenic than cells of other phenotypes[2]. Cancer stem cells are closely associated with tumor initiation, progression, metastasis and even drug resistance. It is currently universally accepted that general chemotherapy is not effective in eliminating cancer stem cells, especially when the tumor becomes resistant. We hypothesized that cancer stem cells could confer tumor resistance to endocrine therapeutic drugs.

In 2003, Michael Clarke's group first identified a CD44^+^, CD24^lo^, ESA^+^ and lineage- subpopulation of human breast cancer cells, which can initiate tumors in immune-deficient NOD/SCID mice[2]. This subpopulation may be defined as cancer stem cells according to the following characteristics: ability for self-renewal, survival from anoikis, high tumorigenic capacity and ability to efflux toxins efficiently[3],[4]. Fillmore *et al*.[5] demonstrated that breast cancer cell lines also contain a stem-like subpopulation based on tumorigenicity *in vivo*. Clinical evidence with neoadjuvant therapy also indicated that these breast cancer stem cells can be selected by chemotherapy rather than by lapatinib[6]. Therefore, drug resistance to chemotherapy is considered as an intrinsic characteristic of breast cancer stem cells.

Although some controversies remain, many researchers believe that cancer stem cells are responsible for resistance to endocrine therapy. The response to endocrine therapy depends on the expression of estrogen receptor (ER) α. Lindeman and his colleagues[7] reported that normal murine mammary stem cells are negative for ERα, progesterone receptor (PR) and erbB2 during breast development. Smalley *et al*.[8] also showed by gene profiling and *in vivo* functional studies of ER-expressing mouse mammary cells that ERα-positive cells are not stem cells. In several *in vitro* tamoxifen resistance models, ERα was downregulated while erbB2 was upregulated as resistance developed[9],[10], which could be reversed by inhibiting the epidermal growth factor receptor (EGFR)/erbB2 signaling pathway[11]. It was also proven that enhanced EGFR/erbB2 signaling in tamoxifen resistant breast cancer cells potentially results from selection for a more stem-like phenotype[6],[12]. Using a three-dimensional clonogenic assay of tumor cells, Sartorius' group determined that ER^–^, PR^–^, CD44^+^ and CK5^+^ cells should be defined as breast cancer stem cells according to their capacity to produce more differentiated cells, the majority of which are ER^+^, PR^+^, CK^–^[13]. In a review of previous studies, Clarke[14] proposed that ER^–^, PR^–^, and CD44^+^ CD24^–^/low cells in breast cancer have the same characteristic of tumorigenic breast cancer stem cells. Because tamoxifen only inhibits the proliferation of estrogen-related breast cancer cells, breast cancer stem cells may be resistant to tamoxifen and survive after treatment.

By sorting the cancer stem cell subpopulation in MCF-7 and its tamoxifen-resistant cell line, TAM-R, we demonstrated that this subpopulation was upregulated in TAM-R cells. We also observed several other characteristics of these cell lines and their subpopulations *in vitro* during our effort to decipher the relationship between breast cancer stem cells and resistance to tamoxifen. In the current study, we sought to determine whether STAT3 mediated tamoxifen resistance of breast cancer stem cells *in vitro*.

## MATERIALS AND METHODS

### Reagents

Fetal bovine serum (FBS), Lipofectamine 2000 and Opti-MEM reduced-serum medium were purchased from Invitrogen (Carlsbad, CA, USA). Iscove's Modified Dulbecco's Medium (IMDM) with phenol red was purchased from Hyclone (Logan, UT, USA). Tamoxifen citrate salt was purchased from Sigma (St. Louis, MO, USA). Fluorescein isothiocyanate (FITC) anti-human CD24 and phycoerythrin (PE) anti-mouse/human CD44 were purchased from eBioscience(San Diego, CA, USA). Rabbit anti-human STAT3 IgG, rabbit anti-human phospho-STAT3 IgG and HRP-goat anti-rabbit IgG were purchased from Cell Signaling Technology (Beverly, MA, USA). Mouse anti-human GAPDH IgG and HRP-goat anti-mouse IgG were purchased from MultiSciences Biotech Co., Hangzhou, China. Cell Counting Kit-8 (CCK-8) was from Dojindo Laboratories (Xiongben County, Kyushu, Japan). The following reagents were from KeyGEN, Nanjing, China: Phosphoprotein extraction kit, BCA protein concentration kit, Super ECL system, and RIPA lysis buffer. Annexin V-FITC apoptosis detection kit was purchased from Bender MedSystems GmbH (Vienna, Austria). STAT3 siRNA oligonucleotides were from GenePharma, (Shanghai, China).

### Cell culture

MCF-7 and TAM-R cell lines were gifts from the laboratory of Dr. Santen at the University of Virginia and cultured in complete medium (CM), which consisted of IMDM supplemented with 5% FBS, 10^5^ U/L penicillin and 100 g/L streptomycin at 37°C with 5% CO_2_. Subsequently, 0.01% methanol and 1 µmol/L tamoxifen citrate salt (dissolved in methanol) were added to MCF-7 and TAM-R cells, respectively. Logarithmically growing MCF-7 and TAM-R cells were cultured in serum free IMDM for 24 h. The cells were then collected and plated in 96-well plates at a density of 8×10^3^ cells per well in 200 µL serum-free CM to detect the IC_50_ of adriamycin by using the CCK-8 kit. After 24 h, the medium was replenished, and two-fold dilutions of adriamycin were added in triplicate at six concentrations, with 4 mg/L as the highest concentration. Methanol (0.01%) was added as vehicle controls. After 24 h, the medium was replenished with CM (100 µL/well), and 10 µL CCK-8 reagent was added in each well. The plates were returned to standard cell incubating conditions for 1 h before colorimetric analysis. The data was analyzed using the following formula: suppression rate = (1-OD_ADM_/OD_control_)×100%. Based on the flow cytometric assays and Western blotting assays, we adjusted cell density to 10^6^ cells/L and plated 2 mL CM per well in 6-well plates. Three d before detection, 0.01% methanol or 1 µmol/L tamoxifen citrate salt was added into the plates. For mammosphere formation assay, the CD44^+^CD24^–/low^ subpopulation of MCF-7 cells, which were selected by FACS, was cultured overnight[15] in serum free medium consisting of DMEM/F12 (1:1) with 5 U/L insulin, 20 U/L EGF and 10 U/L bEGF[16]. Both MCF-7 and CD44^+^CD24^–/low^ MCF-7 cells were collected and plated in 96-well plate at a density of 100 cells per well. Briefly, the cells were suspended in the serum free medium, counted after trypan blue staining for viable cells, and after dilution to a density of 10^6^ cells/L, they were plated in 96-well plate at 100 µL/well. After 72 h, the cells were observed under a microscope and all 96 wells were labeled as positive (+) or negative (-) according to their mammosphere formation status. The mammosphere formation rate was calculated with the following equation: mammosphere formation rate = (1 - number of positive wells/ number of negative wells)×100%. CD44^+^CD24^–/low^ cells were selected by FACS and cultured as described previously[15] in the serum free medium overnight before protein extraction.

### Flow cytometry

Cells were harvested and then distributed at 10^6^ cells/tube. The cells were centrifuged at 2,000×g for 10 min, and the supernatants were removed. For CD44^+^CD24^–/low^ subpopulation analysis, the cells were resuspended in 80 µL PBS, 1 µL CD44-FITC and 10 µL CD24-PE and incubated at 37°C for 1 h. Then, each tube was washed with PBS 3 times and finally resuspended in 400 µL cold PBS. For sorting of CD44^+^CD24^–/low^ subpopulation, 10^7^ cells were treated in a 10 times larger 10^6^ cells system. For apoptosis analysis, cells were treated using the Annexin V-FITC apoptosis detection kit. Briefly, cells were washed with PBS and then resuspended in 195 µL binding buffer (1×). Then, 5 µL annexin V-FITC was added to the cell suspension. After incubation at room temperature for 10 min, cells were washed, followed by resuspension in 190 µL binding buffer and addition of 20 µL propidium iodide (20 mg/L). Based on cell cycle analysis, the cells were resuspended in 400 µL of 75% cold ethanol and incubated at -20°C for at least 12 h. All cells were analyzed using a FACS Calibur flow cytometer (Becton Dickinson, San Jose, CA, USA).

### Immunofluorescence assays

Logarithmically growing cells were fixed with 4% paraformaldehyde and permeabilized with 0.4% Triton X-100. After blocking with PBS/2%BSA, cells were incubated with anti-CD44 antibodies followed by incubation with goat anti-mouse IgG Alexa Fluor 594. Following an additional wash, DAPI was added to cells for staining nuclei.

### Western blotting assays

Cells were homogenized in lysis buffer in the presence of a cocktail of 1% (*V*/*W*) protease inhibitors. After shaking at 4°C for 1 h, the lysates was clarified by centrifugation at 15,000 *g* for 30 min at 4°C. Phosphoproteins were extracted and all protein concentrations were determined using the BCA method according to the manufacturer's instructions. Prior to Western blotting, 5×loading buffer was added to protein samples and rehydrated. The proteins (30 ng per well) were then loaded onto an SDS-PAGE gel. The PVDF membranes onto which the resolved proteins had been transferred were immunoblotted with mouse monoclonal antibodies to STAT3 or phospho-STAT3. HRP goat anti-mouse IgG was used as secondary antibody. Bound antibodies were visualized using the Super ECL system. Densitometric analysis was performed using Quantity One 4.62 (Bio-Rad, Hercules, CA, USA).

### STAT3 small interference RNA studies

STAT3 small interference RNA (siRNA) oligonucleotides, STAT3 siRNA-1 and STAT3 siRNA-2, and corresponding scrambled siRNA oligonucleotides, scrambled siRNA-1 and siRNA-2 (***Table 1***) were transfected into 50% confluent TAM-R cells in 6-well culture plates. The siRNA oligonucleotides and Lipofectamine 2000 were 1:50 diluted in RNase-free water, respectively. Then, they were 1:1 mixed at a final concentration of 0.1 µm siRNA oligonucleotides. The cells were incubated at 37°C for 4 h before addition of CM and cultured for an additional 72 h. Thereafter, the proteins of all these cells were extracted and analyzed by Western blotting. For tamoxifen sensitivity assay, TAM-R cells were plated in 96-well plates. The experimental groups (Exp group) were treated by siRNA (or Lipofectamine 2000 only as a blank control) and tamoxifen (10^–6^ mol/L) together; however, the negative control groups (Con groups) were only treated by siRNA (or Lipofectamine 2000 only as the blank control). After treatment for 72 h, CCK-8 assay was performed to obtain OD values in different groups. Tamoxifen inhibition rates were then calculated [Tamoxifen inhibition rate = (1-OD_Exp_/OD_Con_)×100%].

**Table 1:** Sequences of STAT3 siRNA and scrambled siRNA oligonucleotides

Gene		Sequence (5′-3′)	GC content		Position
STAT3	Sense	AGUCUUUGUCAAUGCACACTT	38.10%		1096 of ORF
siRNA-1	Anti-sense	GUGUGCAUUGACAAAGACUTT			
STAT3	Sense	AUCAAAGUCAUCCUGGAGATT	42.11%		500 of ORF
siRNA-2	Anti-sense	UCUCCAGGAUGACUUUGAUTT			
Mock	Sense	UCAUGUAUCAGUCAUCACGTT		Non	
siRNA-1	Anti-sense	CGUGAUGACUGAUACAUGATT		
Mock	Sense	AGCUUGAUACGACAAAGCUTT		Non	
siRNA-2	Anti-sense	UCGAACUAUGCUGUUUCGATT		

### Statistical analysis

All the data were analyzed by SPSS 13.0 software (SPSS Inc., Chicago, IL, USA). For the data of STAT3 siRNA experiment, the variance was analyzed firstly. If the variance was heterogenic, both Chi-square test and Rank test were performed. Other data were analyzed by independent sample *t*-test. Statistical significance was accepted at *P* < 0.05.

## RESULTS

### The CD44^+^CD24^−/low^ subpopulation represents cancer stem cells in MCF-7 cell line

**Fig. 1:**
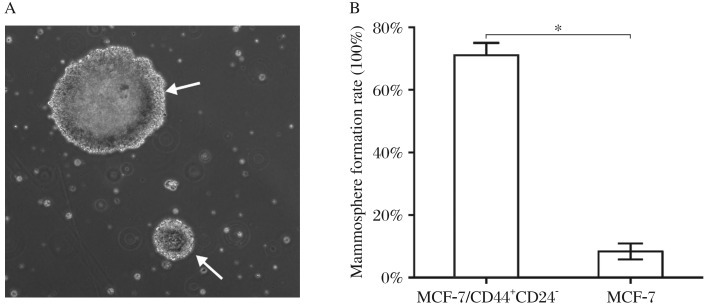
Mammosphere formation assay. A: After 72 h of culture in serum free medium (SFM), mammospheres (indicated by white arrow) were formed by CD44^+^CD24^−/low^ MCF-7 cells (magnification 100×). B: CD44^+^CD24^−/low^ subpopulation of MCF-7 cells (cancer stem cells, CSCs) selected by FACS were pre-cultured in SFM overnight. Subsequently, both MCF-7 and CD44^+^CD24^−/low^ MCF-7 cells were collected and plated in 96-well plates at a density of 100 cells per well. After 72 h, all wells were observed under a microscope and labeled as positive (+) or negative (-) according to their mammosphere formation status. There was a significant difference in mammosphere formation rates between CD44^+^CD24^−/low^ MCF-7 cells and MCF-7 cells (*t*-test, *P < 0.05).

In the mammosphere formation assay, both MCF-7 cells and its CD44^+^CD24^−/low^ subpopulation could form mammospheres after 72 h culture in the serum free medium (***Fig. 1A***), although they had different mammosphere formation rates, 71.11±6.74% vs. 8.33±4.41% (*T*-test, *P* < 0.05, ***Fig. 1B***), indicating that the CD44^+^CD24^–/low^ subpopulation of MCF-7 cells achieved at least a 7-fold greater mammosphere formation capacity than MCF-7 cells. It can be inferred that the CD44^+^CD24^–/low^ subpopulation represents cancer stem cells in MCF-7 cell line.

### Relationship between cancer stem cells and tamoxifen resistance

**Fig. 2:**
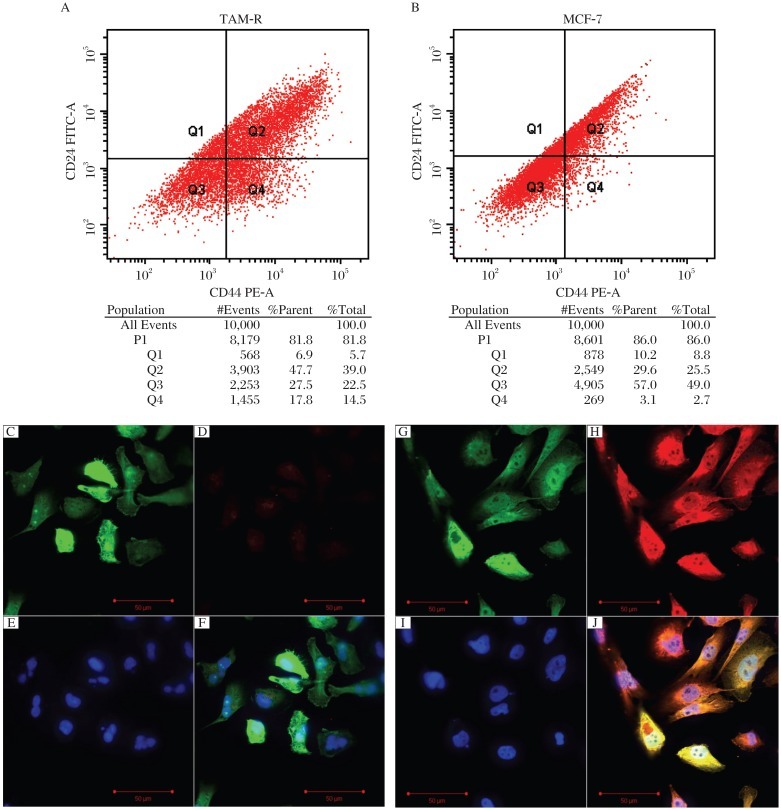
CD44^+^CD24^−/low^ subpopulation in MCF-7 and TAM-R cells. The CD44^+^CD24^−/low^ subpopulation of TAM-R cells was (A) higher than that in MCF-7 cells (B) detected by FACS (*P* < 0.05). CD44 expression in TAM-R cells was much higher than that of MCF-7 cells detected by immunofluorescence. C and G: GFP expression of cells; D and H: CD44 expression (stained by PE); E and I:nucleus stained by DAPI; F: all of C, D, and E in one image; J: all of G, H, and J in one image. The C, D, E, and F images showed the results of MCF-7 cells and the G, H, I, and J showed the results of TAM-R cells.

**Fig. 3:**
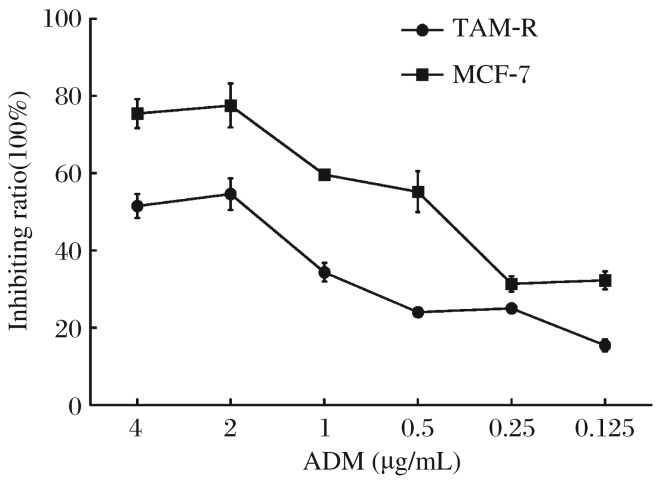
Suppression of proliferation of MCF-7 and TAM-7 cells by ADM detected by a CCK-8 assay. Logarithmically growing MCF-7 and TAM-R cells were cultured in serum free IMDM for 24 h. Both were collected and plated in 96-well plates at a density of 8×10^3^ cells per well. After 24 h, the medium was changed and two-fold dilutions of ADM at six concentrations starting at 4 µg/mL were added and the experiment was done in triplicate. Methanol (0.01%) was added to another set of triplicate wells as controls. After 24 h, the medium was replenished, and CCK-8 reagent was added in each well. The plates were returned to standard cell incubator conditions for an additional h. Colorimetric analysis was then performed, and the suppression rate was calculated. IC_50_ was calculated based on the suppression rate curve (1.37±0.043 µg/mL and 1.71±0.062 µg/mL, respectively, *P* < 0.05). ADM: adriamycin.

FACS analysis showed that the CD44^+^CD24^–/low^ subpopulation ratio in TAM-R cells was much higher than that in MCF-7 cells (*P* < 0.05, ***Fig. 2A***), and there was no significant difference in CD24^–^ subpopulation between TAM-R and MCF-7 cells. Therefore, we used an immunofluorescence assay to detect and confirm CD44 expression in these cells, and we found that TAM-R cells expressed a much higher level of CD44 than MCF-7 cells (***Fig. 2B***). As chemoresistance is an intrinsic characteristic of cancer stem cells, the IC_50_ of adriamycin in MCF-7 and TAM-R cells was determined, which stood at 1.37±0.043 µg/mL and 1.71±0.062 µg/mL, respectively (*P* < 0.05, ***Fig. 3***), indicating that acquired tamoxifen resistance might be associated with breast cancer stem cells, which could contribute resistance to general chemotherapy.

### STAT3 may mediate resistance of cancer stem cells to tamoxifen

**Fig. 4:**
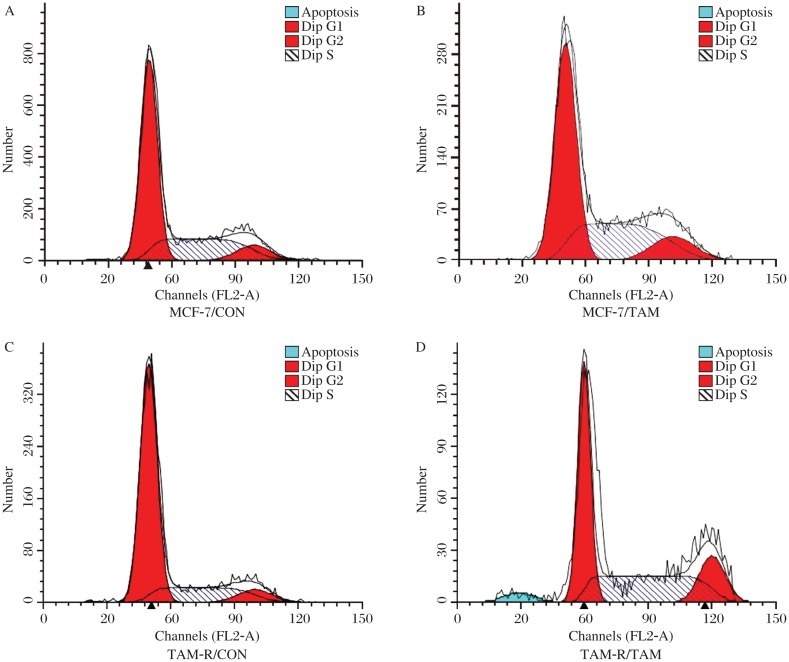
Cell cycle changes in MCF-7 and TAM-R cells by tamoxifen (TAM). After treatment with 0.01% methanol or 10^−6^ mol/L TAM citrate salt for 3 d, changes in cell cycle distribution were detected by FACS. The upregulation rate of S phase in the table was calculated by the formula as follows, Up-Rate = (S phase ratio TAM / S phase ratio Con)×100%. There was a significant change in cell cycle distribution with an upregulation of S-phase after TAM treatment in MCF-7 cells but not in TAM-R cells (*P* < 0.05). A: MCF-7/CON: MCF-7 cells treated with 0.01% methanol; B: MCF-7/TAM: MCF-7 cells treated with TAM; C: TAM-R/CON: TAM-R cells treated with 0.01% methanol; D: TAM-R/TAM: TAM-R cells treated with TAM.

**Fig. 5:**
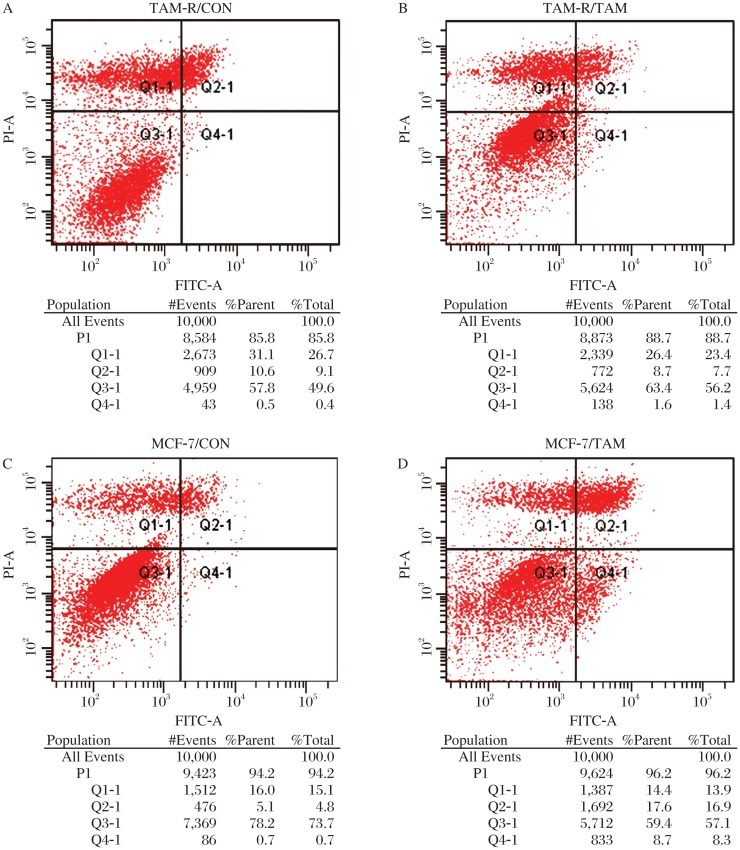
Apoptotic changes in tamoxifen (TAM) treated MCF-7 and TAM-R cells. After treatment with 0.01% methanol or 10^−6^ mol/L TAM citrate salt for 3 d, cell apoptosis was detected by FACS; The change rate of apoptosis ratio in the table was calculated by the formula as follows, Ch-Rate = TAM/CON×100%. There was a significant upregulation of apoptosis after TAM treatment in MCF-7 cells but not in TAM-R cells (*T*-test, *P* < 0.05). A: MCF -7/CON: MCF -7 cells treated with 0.01% methanol; B: MCF-7/TAM: MCF -7 cells treated with TAM; C: TAM-R/CON: TAM-R cells treated with 0.01% methanol; D: TAM-R/TAM: TAM -R cells treated with TAM.

**Fig. 6:**
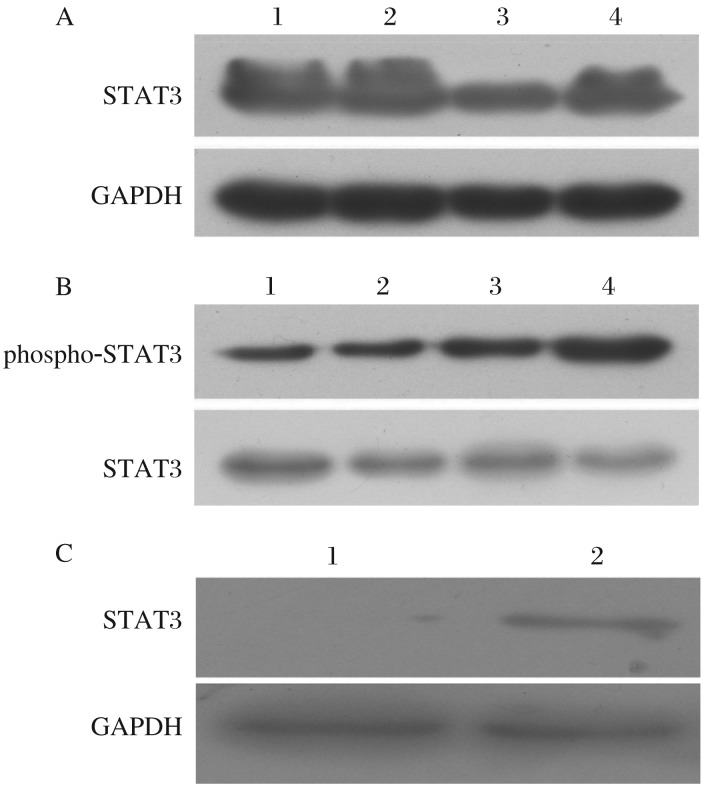
STAT3 and phospho-STAT3 expression detected by Western blotting. A: Expressions of STAT3 was determined by Western blotting in MCF-7 and TAM-R cells treated with or without tamoxifen (TAM). lane 1: TAM-R; lane 2: TAM-R/TAM;lane 3: MCF-7; lane 4: MCF-7/TAM. Significant upregulation of STAT3 in TAM-R and TAM treated TAM-R or MCF-7 cells was observed. B: Phospho-STAT3 was shown to be significantly upregulated in TAM treated TAM-R cells; lane 1: MCF-7; lane 2: MCF-7/TAM; lane 3: TAM-R; lane 4: TAM-R/TAM. C: STAT3 detected by Western blotting was upregulated in FACS sorted CD44^+^CD24^−/low^ MCF-7 cells compared with MCF-7 cells. lane 1: MCF-7; lane 2: MCF-7/CD44^+^CD24^–^. In A and B, MCF-7: MCF-7 cells treated with 0.01% methanol; MCF-7/TAM: MCF-7 cells treated with TAM; TAM-R: TAM-R cells treated with 0.01% methanol; TAM-R/TAM: TAM-R cells treated with TAM. In C, MCF-7/CD44^+^CD24^−/low^: CD44^+^CD24^−/low^ MCF-7 cells.

After treatment with 0.01% methanol or 10^−6^ mol/L TAM citrate salt for 3 d, changes in cell cycle distribution and cell apoptosis were both detected by FACS (***Fig. 4*** and ***Fig. 5***). There was a significant change in cell cycle distribution with an upregulation of S-phase after TAM treatment in MCF-7 cells but not in TAM-R cells (*P* < 0.05, ***Fig. 4***). Also, there was a significant upregulation of apoptosis after TAM treatment in MCF-7 cells but not in TAM-R cells (*P* < 0.05, ***Fig. 5***). Compared to MCF-7 cells, both upregulation of proliferation (***Fig. 4***) and downregulation of apoptosis (***Fig. 5***) were associated with tamoxifen resistance of TAM-R cells *in vitro*. Meanwhile, cancer stem cells also play a role in resistance to tamoxifen. Therefore, we detected STAT3 to determine whether it was one of the regulatory pathways mediating this process. Compared to MCF-7 cells, STAT3 was upregulated in TAM-R cells and tamoxifen-treated TAM-R or MCF-7 cells (***Fig. 6A***). Meanwhile, phospho-STAT3 was upregulated in TAM-R and tamoxifen-treated TAM-R cells with a higher up-regulation in TAM-R cells, which was not observed in MCF-7 and tamoxifen-treated TAM-R cells (***Fig. 6B***). We also observed that STAT3 expression in cancer stem cells (CD44^+^CD24^–/low^ subpopulation) of MCF-7 cells was much higher than that in MCF-7 cells. All these results indicated that STAT3 might mediate tamoxifen resistance of cancer stem cells.

**Fig. 7:**
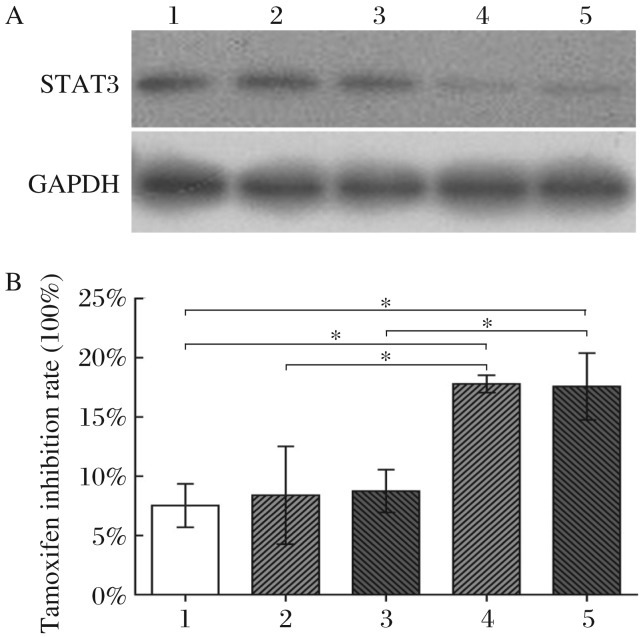
STAT3 knockdown by siRNA sensitizes TAM-R cells to tamoxifen (TAM). A: STAT3 expression in STAT3 siRNA or scrambled siRNA-treated TAM-R cells was identified by Western blotting. B: After successful knockdown of STAT3, as described in Methods, TAM sensitivities of TAM-R, scrambled siRNA-treated TAM-R and STAT3 siRNA-treated TAM-R cells were detected using the CCK-8 assay. Compared to TAM-R cells, the TAM inhibition rate of STAT3 siRNA-treated TAM-R cells was upregulated in parallel with STAT3 expression in the drug resistant phenotype of cancer stem cells. Group 1, 2, and 4 were analyzed by Chi-square test and Rank test (Heterogeneity of variance), group 1 *vs* group 2, *P* > 0.05, and group 1 *vs* group 4 or group 2 *vs* group 4, *P* < 0.05. Group 1, 3, and 5 were analyzed by Chi-square test (Homogeneity of variance), group 1 *vs* group 3, *P* > 0.05, and group 1 *vs* group 5 or group 3 *vs* group 5, *P* < 0.05. 1: TAM-R cells treated with only Lipofectamine 2000; 2: TAM-R cells treated with scrambled siRNA-1; 3: TAM-R cells treated with scrambled siRNA-2; 4: TAM-R cells treated with STAT3 siRNA-1; 5: TAM-R cells treated with STAT3 siRNA-2; *P < 0.05.

Based on these findings, siRNA experiments were performed to downregulate STAT3 expression in TAM-R cells (***Fig. 7A***). A consistent upregulation of tamoxifen sensitivity after treatment with STAT3 siRNA was also observed in TAM-R cells (***Fig. 7B***). Comparison of the group of scrambled siRNA-treated TAM-R cells with Lipofectamine 2000-treated TAM-R cells revealed no statistical significance (*P* > 0.05). However, comparison of STAT3 siRNA-treated TAM-R cells with Lipofectamine 2000-treated TAM-R cells or scrambled siRNA-treated TAM-R cells revealed a statistically significant difference (*P* < 0.05).

## DISCUSSION

In this study, we examined the mechanism of tamoxifen resistance of CD44^+^CD24^–/low^ breast cancer stem cells *in vitro*. Consistent with the observations of drug resistance to chemotherapy in breast cancer, our study showed that cancer stem cells contribute to tamoxifen resistance *via* exerting anti-apoptotic effects and counteracting cell cycle changes caused by tamoxifen. Importantly, we find that STAT3 in the JAK-STAT signaling pathway may partially mediate the resistance of breast cancer stem cells to tamoxifen.

In 2008, Fillmore *et al*.[5] examined the presence of cancer stem cells in breast cancer cell lines. For all the cell lines in their study, including those possessing a mesenchymal phenotype such as MDA-MB-231, the percentages of CD44^+^CD24^–/low^ESA^+^ cells rather than CD44^+^CD24^–/low^ cells within the cell lines correlated with tumorigenicity, self-renewal, reconstitution of the parental cell line, retention of BrdU labeling and preferential survival from chemotherapy. The epithelial special antigen (ESA) is an important marker for sorting cancer stem cells in breast cancer cell lines, and it is broadly expressed in epithelial cells like MCF-7 cell line. Therefore, CD44^+^CD24^–/low^ as the specific marker for stem-like cell population in MCF-7 cells has been used broadly[17],[18]. At present, there are two conventional methods to identify cancer stem cells in solid tumors. One method is comparing tumorigenic capacities of breast cancer stem cells and their parental cells in xenograft nude mice, which was first used by Clark and his colleagues in 2003[2]. The other one was a breast cancer mammosphere formation assay designed by Dontu *et al*. It was accepted as an *in vitro* method to identify cancer stem cells[16]. In our study, CD44+CD24–/low cells of MCF-7 had a higher mammosphere formation rate than MCF-7 cells. For the *in vitro* acquired tamoxifen resistance model, the percentage of CD44^+^CD24^–/low^ cells was upregulated in TAM-R cells. Significantly, TAM-R became resistant to chemotherapy, which is recognized as an intrinsic characteristic of breast cancer stem cells, at the same time they acquired resistance to endocrine therapy. Compared to MCF-7, both the upregulation of CD44^+^CD24^–/low^ subpopulation ratio and IC_50_ of adriamycin indicated that breast cancer stem cells displaying chemoresistance would also play an important role in tamoxifen resistance.

Due to its effectiveness (70% response rate in ER-positive tumors), such as lack of severe toxicity compared with cytotoxic chemotherapeutic agents, beneficial effects against osteoporosis and coronary vascular disease, tamoxifen is broadly used as a therapeutic agent for hormone responsive breast cancer[19],[20]. It is also a chemo-preventative agent for women who have a familial history of breast cancer[21]. The clinical efficacy of tamoxifen has been proven to be for both growth arrest and induction of apoptosis within breast cancer cells. A previous *in vitro* study has also demonstrated that tamoxifen can induce apoptosis of MCF-7 cells[22]. In the therapy of breast cancer, patients receive tamoxifen daily for at least 3 months, and Dixon's group[23],[24] demonstrated that clinical response to tamoxifen is associated with increased apoptosis and decreased proliferation of breast cancer cells by detecting surrogate markers of apoptosis (Bcl-2) and mitosis (Ki-S1).

After analyzing both the 4-hydroxy and *N*-desmethyl metabolites of tamoxifen, Fabian *et al.*[25] found that their ER-binding affinities were higher than or equal to those of tamoxifen. Mandlekar *et al.*[26] later proved that both metabolites are able to induce apoptotic cell death in ER-positive MCF-7, ER-negative MDA MB 231 and BT-20 breast cancer cells. These results indicated that induction of apoptosis could be a major mechanism of the anti-tumor effect of tamoxifen. Now, we observed that tamoxifen could induce apoptosis in both MCF-7 and TAM-R cells, but the apoptosis level was much lower in TAM-R cells. In spite of the differences in anti-tamoxifen induced apoptosis and the proportions of cancer stem cell subpopulation between MCF-7 and TAM-R cells, we inferred that the mechanism of breast tumorigenesis by cancer stem cells may be related to an anti-apoptosis effect and, consequently, tamoxifen resistance. Both genomic nuclear-initiated estrogen signaling (NIES) mediated by ER-α66 and non-genomic membrane-initiated estrogen signaling (MIES) mediated by non-ER-α66 or other signaling pathways participate in the anti-tumor effect of tamoxifen. The signaling proteins in the latter include protein kinase C (PKC)[27], TGF-β[28], calmodulin[29], c-myc[30], ceramide[31] and MAP kinases[32].

Seven members of the STAT family have been cloned (STAT1∼4, 5a, 5b, and 6), among which STAT5a and STAT3 were confirmed to be most strongly associated with the proliferation and oncogenesis of human breast cancer cells. STAT3 activation can upregulate the expression of anti-apoptotic proteins (Bcl-2, Mcl-1 and Bcl-x), proliferation related proteins (cyclin D1 and c-Myc) and angiogenesis promoting factors (VEGF) to prevent tumor cells from apoptosis[33]. Recently, using the mammosphere model combined with DNA methylation bead arrays and quantitative gene expression analysis, Hernandez-Vargas *et al*. demonstrated a constitutive activation of the JAK-STAT pathway in CD44+CD24-/low breast cancer stem cells[34]. This result indicated that Jak-STAT activation may be an intrinsic characteristic of breast cancer stem cells. As early as 1997, Sartor *et al*.[35] found that autonomous proliferation of breast cancer cell lines is driven by the STAT3 signaling pathway, which is related to EGFR. Thereafter, Bromberg *et al*.[36] demonstrated that a constitutively activated STAT3 mutant alone is sufficient to induce transformation and tumor formation in nude mice. In studying the mechanism of STAT3 activation that induced tumorigenesis of breast tissue, Berclaz *et al*.[37] found that the tyrosine kinase signaling pathway plays an important role in the transformation of breast tissue, which includes ER co-activators such as AIB1 and c-Src that contribute to tamoxifen resistance[38],[39]. Even in breast cancer cell lines, including MCF-7 and MDA-MB-231, STAT3 was previously shown to be associated with cell proliferation[40]. Furthermore, activated by the interaction between hyaluronan and CD44, the stem cell specific marker Nanog forms a complex with STAT3 in the nucleus, leading to STAT3-specific transcriptional activation and multidrug transporter MDR1 (P-glycoprotein) gene expression, which mediates chemoresistance (e.g. Adriamycin and Taxol)[41].

Detailed insights into the role of STAT3 in tumor development, progression and drug resistance directly point to new specific targeting strategies for tumor therapy. Several therapeutic strategies directing at STAT3 have been developed, which focus on the anti-tumor effect only. For instance, a 28-mer peptide, SPI, derived from the STAT3 SH2 domain, can be used as a selective inhibitor of STAT3 activation with antitumor cell effects[42]. In our study, STAT3 was implicated as a mediator of tamoxifen resistance in breast cancer stem cells. These results highlight that STAT3 is a strong candidate target not only for anti-tumor therapy, but also for tamoxifen resistance in treatment with breast cancer. apart from its general anti-proliferative effects, strategies targeting the STAT3 signaling pathway can directly inhibit breast cancer stem cells and control tumorigenesis at its source. As well as becoming a prognostic marker, the well-studied STAT3 pathway can therefore be targeted by a number of inhibition strategies at different levels in cancer therapy.
